# Measurement Bias in Documentation of Social Risk Among Medicare Beneficiaries

**DOI:** 10.1001/jamahealthforum.2025.1923

**Published:** 2025-07-18

**Authors:** Paula Chatterjee, Eliza Macneal, Eric T. Roberts

**Affiliations:** 1 Department of Medicine, University of Pennsylvania Perelman School of Medicine, Philadelphia; 2 Leonard Davis Institute of Health Economics, University of Pennsylvania, Philadelphia

## Abstract

**Importance:**

Health care organizations are increasingly measuring social risk using Z codes. Types of social risk captured in Z codes include issues related to employment, housing, education, or other psychosocial circumstances. Prior work has found low use of Z codes overall, but measurement may be biased in other ways that have implications for risk adjustment and resource allocation.

**Objective:**

To characterize Z code measurement among hospitalized Medicare beneficiaries across levels of clinical complexity and historical health care utilization and examine implications of these patterns for mortality prediction.

**Design, Setting, and Participants:**

This retrospective cohort study included Medicare beneficiaries with an inpatient hospital admission in 2022. Data were analyzed from May 2024 to June 2025.

**Main Outcomes and Measurements:**

Presence of Z codes (codes Z55 to Z65) in any diagnosis field for a hospital admission, variation in Z code documentation across beneficiaries categorized by clinical risk (Elixhauser Comorbidity Index risk scores and predicted 30-day mortality risk) and historical utilization levels (number of hospitalizations in the prior year), and the association between Z code documentation and observed 30-day mortality, controlling for hospital fixed effects.

**Results:**

Among 7 069 611 hospitalized Medicare beneficiaries in 2022, 3 816 420 (54.0%) were female, and 6 093 932 (86.1%) were 65 years or older. A total of 148 592 (2.1%) had at least 1 Z code on the index hospital claim. Within-hospital Z code prevalence was higher for beneficiaries with lower Elixhauser Comorbidity Index clinical risk scores (2.8% vs 1.5%) and higher among patients with at least 2 hospitalizations in the prior year (2.6%) than patients with zero (1.8%) or 1 (2.1%) prior hospitalizations. Despite known population-level associations between social risk and increased mortality, Z code prevalence was highest among beneficiaries with the lowest predicted 30-day mortality risk (4.4%) and lowest among beneficiaries with the highest mortality risk (1.6%). Correspondingly, in within-hospital analyses that did not adjust for patient-level covariates such as demographic characteristics and clinical risk, the presence of a Z code was associated with a lower probability of observed 30-day mortality (5.1% vs 4.2%; difference, −0.9 percentage points; 95% CI, −1.0 to −0.8).

**Conclusions and Relevance:**

This cohort study found that Z code use patterns likely underrepresent social risk among clinically complex patients, resulting in a spurious negative association between documented social risk and mortality. Alternative socioeconomic indicators, including data collected for population and public health surveillance, may offer more reliable measures of social risk than Z codes.

## Key Points

**Question:**

Are documentation patterns for Z codes, which are used to capture social risk, susceptible to measurement bias relative to clinical complexity and historical health care utilization?

**Findings:**

In this cohort study of more than 7 million hospitalized Medicare beneficiaries in 2022, Z codes were used more often for less clinically complex patients and patients with higher levels of historical health care utilization. These patterns lead to a spurious negative association between documented social risk and predicted mortality.

**Meaning:**

Existing documentation patterns for social risk are susceptible to measurement bias through multiple mechanisms and have important implications for risk adjustment, value-based payment, and health care resource allocation.

## Teaser

This cohort study characterizes Z code measurement among hospitalized Medicare beneficiaries across levels of clinical complexity and historical health care utilization and examines implications of these patterns for mortality prediction.

## Introduction

Health care organizations are increasingly measuring social risk among patients they serve.^1,2,3^ The *International Statistical Classification of Diseases and Related Health Problems, Tenth Revision *(*ICD*-*10*) introduced a set of diagnosis codes to document social risk in the medical record, termed *Z codes*.^4^ The types of social risk captured in Z codes include issues related to employment, housing, education, or other psychosocial circumstances. To date, Z codes primarily have been used to document health-related social needs but have not been used to risk adjust quality measures or had an extensive impact on physician and hospital payment. Accordingly, uptake of Z codes has been slow, and their use in inpatient settings has been limited and concentrated among patients with mental health or psychiatric conditions.^5,6,7^

The low overall use of Z codes raises concerns that they may inadequately capture the true burden of social risks. In addition to underuse, Z code measurement may be biased in other ways. First, documentation of Z codes could vary systematically among patients. For example, Z codes may be documented less frequently for clinically complex patients if greater attention is placed on medical rather than social risk during hospitalization. This may result in undercoding of social risk because clinically complex patients are often more likely to have social risk factors that lead to delayed and severe presentations of disease.^8,9,10,11^ Second, relying on health care utilization to document social risk may limit opportunities to document Z codes for groups with limited access to care.^12^ This may lead to underreporting of social risk among patients who, in reality, have substantial social needs. In these ways, the differential use of Z codes by level of clinical risk or health care utilization may lead to inaccurate assessments of social risk. Such measurement bias could affect the prediction of clinical outcomes, such as readmission or mortality, when linked to social risk documented on the medical record.^13,14^ Measurement bias in Z codes could also shape how organizations monitor and address social risk factors and affect finances of health care organizations if Z codes are integrated into payment policies.

Understanding measurement bias in large-scale data sources is crucial for ensuring that data are used to draw appropriate inferences. Without this understanding, mismeasurement of social risk may lead to misallocation of resources and policy attention. In this study, we investigated patterns of Z code use among hospitalized Medicare beneficiaries. First, we described the relationship between documentation of Z codes on inpatient claims and geographic and individual-level correlates of social risk. Second, we examined whether Z code documentation varied by beneficiaries’ clinical complexity and historical utilization. Third, we examined the association between the presence of Z codes and predicted 30-day mortality risk. These analyses shed light on patient subpopulations for whom social risk may be underrecognized.

## Methods

This study was approved by the Institutional Review Board at the Perelman School of Medicine and followed the Strengthening the Reporting of Observational Studies in Epidemiology (STROBE) reporting guideline. Informed consent was waived as this study used deidentified data.

### Overview of Z Codes

Z codes were introduced in October 2015 to document social risk factors in the medical record. Their purpose was manifold and included improving screening and referrals for social conditions associated with poor disease control and outcomes, motivating quality improvement programs, and prioritizing population health goals. Coders could apply Z codes using documentation from any clinical team member (including nurses, social workers, and case managers), and Z codes could be documented in outpatient, inpatient, and postacute settings. Since introduction, Z codes have been infrequently documented; the prevalence of Z codes documented across health care encounters was estimated at 1.4%, with more common reporting among patients who were young, male, publicly insured, and had a history of a mental health condition.^15^

To date, payers have not provided direct reimbursement for reporting Z codes, although outpatient clinicians can bill at higher levels of clinical complexity if Z codes are documented during a visit. As of January 1, 2024, hospitals are required to report screening rates for a subset of social determinants. Additionally, 2 new inpatient quality indicators from the US Centers for Medicare & Medicaid Services assess performance on social risk screening.^16,17^

### Data and Sample

We analyzed a 100% sample of hospitalized Medicare beneficiaries (with either traditional Medicare or Medicare Advantage) in 2022. For beneficiaries with multiple hospitalizations, we analyzed their first admission in 2022. We included individuals with 12 months of Medicare coverage before hospitalization who were discharged alive and not with hospice care. To observe outcomes within 30 days of the hospital stay, we limited the sample to patients discharged on or before November 30, 2022.

We used the Master Beneficiary Summary File to assess patients’ demographic characteristics (age, race, ethnicity, and sex), dual eligible status, zip code of residence, and date of death. Race and ethnicity were included because racial and ethnic minority groups have a higher burden of health-related social needs than other groups.^18,19^ We assessed race and ethnicity using the enhanced Research Triangle Institute variable, which draws from Social Security Administration demographic data and uses an imputation algorithm to identify additional Asian and Hispanic beneficiaries.^20^ We linked beneficiaries’ zip codes of residence to 5-year pooled zip code area poverty rates among residents 65 years and older from the 2019 American Community Survey. We identified dual-eligible patients who had full Medicaid coverage, signifying that they had low incomes.^21^ We used dual eligibility status and residence in high-poverty zip codes (highest national quartile of the proportion of individuals 65 years and older living in poverty) as proxies for socioeconomic status that we could measure independently of Z codes.^22,23^

We used the Medicare Provider Analysis and Review file to identify inpatient admissions and information on patients’ treatment hospital, number of diagnoses, Diagnosis Related Group (DRG) codes, and number of hospitalizations in the prior year. We used Elixhauser Comorbidity Software Refined for *ICD-10-CM* to identify comorbidities and calculated an Elixhauser Comorbidity Index score using diagnoses on the index hospital claim and hospital claims over the prior year.^24^ The Elixhauser Comorbidity Index is a weighted sum of comorbidities, where weights are normalized coefficients of a logistic regression model of in-hospital mortality. Because some comorbidities are associated with reduced likelihood of in-hospital mortality, the index range includes negative numbers.^24^

### Statistical Analysis

Our primary outcome was the presence of a Z code documented on the sample admission. We created a binary indicator denoting the presence of a Z code (codes Z55 to Z65) on any diagnosis field of the admission record (eTable 1 in Supplement 1).

We first examined the unadjusted prevalence of Z codes in the full sample and subpopulation strata defined by age (younger than 65 years vs 65 years and older), sex (male or female), race and ethnicity (Hispanic, non-Hispanic Black, non-Hispanic White, or other race [aggregated due to small samples and including Asian or Pacific Islander, American Indian or Alaska Native, other race, and unknown race]), reason for Medicare entitlement (old age, disability insurance benefits), dual eligibility status (fully dual eligible for past 12 months vs non–dual eligible), quartile of zip code poverty rate, and quartile of Elixhauser Comorbidity Index. We chose these strata because health-related social needs are more prevalent among younger people, men, racial and ethnic minority groups, people with disabilities, dual-eligible beneficiaries, people living in low-income areas, and people at high risk of mortality.^5,25^

Next, we examined the association between clinical complexity and presence of a Z code. We hypothesized that clinically complex beneficiaries might be less likely to have Z codes, given the priority for measuring and managing medical risk over social risk in clinically complex contexts. To test this, we fitted linear regressions to model the presence of a Z code as a function of 3 clinical complexity measures: number of diagnoses reported for the index hospitalization, Elixhauser Comorbidity Index score, and DRG code for the hospitalization. Linear regression can be used to model binary outcomes with coefficients interpreted as conditional probabilities of the outcome.^26^ A separate model was estimated for each measure. We categorized patients into quartiles of the number of diagnoses and the Elixhauser Comorbidity Index. We classified the 10 most common DRG codes as either primary general medical, primary mental health and/or disability, or primary surgical. To maintain comparisons among patients with similar levels of socioeconomic disadvantage, in addition to a model for the full sample, we fitted separate models for dual-eligible and non–dual-eligible beneficiaries, and patients living in zip codes in the highest and lowest quartiles of poverty. Each model included hospital fixed effects to account for hospital-level differences in case mix, coding intensity, and documentation practices. We used marginal effects to calculate the average within-hospital association between patient-level clinical complexity and probability of Z code use, holding the effects of specific hospitals constant.

Finally, we examined the association between Z code documentation and mortality. Because increased social risk is linked to increased clinical risk,^8,9,10^ we would expect the presence of a Z code to be positively correlated with mortality risk. However, underuse of Z codes in clinically complex populations might result in a negative correlation. We examined whether the documentation of Z codes was negatively correlated with mortality risk in 2 ways. First, we plotted the proportion of patients with a Z code across ventiles of predicted 30-day postdischarge mortality risk. To predict 30-day postdischarge mortality risk, we fitted an admission-level linear regression model of mortality as a function of individual characteristics (sex, square of age to account for the nonlinear relationship between mortality and age, reason for Medicare entitlement, 38 binary indicators of Elixhauser Comorbidity Index comorbidities, and admission DRG code), and hospital fixed effects. We assigned each patient a risk score based on individual characteristics, excluding the hospital fixed effects.^13^ For comparison, we plotted the association between ventiles of area-level poverty and presence of a Z code, since we expect the presence of Z codes to be positively associated with area-level poverty.

Second, we examined the association between Z codes and observed 30-day postdischarge mortality. We fitted 5 linear admission-level models with 30-day mortality as the outcome and presence of a Z code as the explanatory variable. The base model included hospital fixed effects; subsequent models incrementally adjusted for the square of age, sex, race and ethnicity, and Elixhauser Comorbidity Index score. Lower Z code use for individuals with higher clinical risk could result in a negative correlation between the presence of a Z code and mortality. However, if Z codes are used less frequently for individuals with higher clinical risk, controlling for those clinical factors could attenuate or eliminate this negative correlation.

We conducted 4 supplementary analyses. First, because the frequency of health care use affects opportunities for Z code documentation, we calculated a Spearman rank correlation coefficient (ρ) between hospital-level Z code prevalence and the mean number of diagnoses per admission. This value can range between −1 and 1, with values closer to 1 reflecting a strong positive correlation and values closer to −1 reflecting a strong negative correlation. We then modeled the presence of a Z code as a linear function of a patient having 0, 1, or at least 2 hospitalizations in the prior year. We included hospital fixed effects and used marginal effects to report the within-hospital associations between prior-year hospital utilization and probability of Z code use. We again fitted separate models for dual-eligible patients, non–dual-eligible patients, and residents of zip codes in the highest and lowest quartiles of poverty among older adults.

Second, to assess heterogeneity in Z code prevalence across ventiles of 30-day mortality risk, we stratified plots of Z code prevalence across types of admissions (primary mental health and/or disability admissions, general medical admissions, surgical admissions), and type of Medicare coverage (Fee-for-Service Medicare or Medicare Advantage).

Third, to interrogate whether differences in Z codes were linked to hospital-level coding patterns, we evaluated 2 models. First, we used an admission-level regression to model the presence of a Z code as a function of patient characteristics (the square of age, disability, dual-eligibility status, residence in a high-poverty zip code) and hospital fixed effects. The coefficient associated with each hospital fixed effect centered at the mean reflected the adjusted proportion of admissions with a Z code for a specific hospital. Second, we used a similar admission-level regression to model the number of diagnoses as a function of the same patient characteristics and hospital fixed effects. The coefficient for each hospital fixed effect centered at the mean reflected a hospital’s overall diagnostic coding intensity. We correlated the 2 sets of hospital fixed effects and examined how Z code prevalence varied by level of clinical risk within hospital tertiles based on diagnostic coding intensity.

Fourth, we repeated the main analyses of Z code prevalence across measures of clinical complexity using a patient’s last (instead of first) hospitalization from January to November 2022. Analyses were performed using SAS version 9.4 (SAS Institute). Data were analyzed from May 2024 to June 2025.

## Results

### Sample

The sample included 7 069 611 Medicare beneficiaries with a hospitalization in 2022; 3 816 420 (54.0%) were female, and 6 093 932 (86.1%) were 65 years or older (Table 1).^27,28,29^ A total of 148 592 (2.1%) had a Z code on the index hospital claim. Z code prevalence was higher among beneficiaries younger than 65 years compared with 65 years and older (47 740 [4.9%] vs 100 852 [1.7%]) and among non-Hispanic Black beneficiaries and Hispanic beneficiaries (24 552 [2.9%] and 12 333 [2.2%]) compared with non-Hispanic White beneficiaries (104 869 [2.0%]). Z code prevalence was also higher among beneficiaries who originally qualified for Medicare due to a disability vs age (3.5% vs 1.6%); dual-eligible vs non–dual-eligible beneficiaries (3.7% vs 1.8%); and beneficiaries living in zip codes in the highest quartile of poverty vs those living zip codes in the lowest quartile (2.6% vs 1.7%).

**Table 1.** Medicare Beneficiaries With 1 or More Hospitalizations in 2022, Stratified by Presence of a Z Code in Their First Hospitalization of 2022^a^

Characteristic	Medicare beneficiaries with ≥1 hospitalizations in January-November 2022	
	Full sample, No.	Beneficiaries with Z codes, No. (%)
Total	7 069 611	148 592 (2.1)
Age, y		
<65	975 679	47 740 (4.9)
≥65	6 093 932	100 852 (1.7)
Sex		
Female	3 816 420	78 912 (2.1)
Male	3 253 191	69 680 (2.1)
Race and ethnicity^b^		
Hispanic	563 935	12 333 (2.2)
Non-Hispanic Black	849 167	24 552 (2.9)
Non-Hispanic White	5 306 385	104 869 (2.0)
Other race	350 124	6838 (2.0)
Original reason for Medicare entitlement		
Old age	5 036 592	78 532 (1.6)
Disability insurance benefits	1 925 379	68 162 (3.5)
End-stage kidney disease^c^	107 640	1898 (1.8)
Full dual eligible status		
Fully dual eligible for 12 mo	1 247 841	46 294 (3.7)
Not fully dual eligible for past 12 mo	5 821 770	102 298 (1.8)
Medicare enrollment status		
Traditional Medicare	2 915 151	61 605 (2.1)
Medicare Advantage	4 154 460	86 987 (2.1)
Quartile of zip code poverty rate^d^		
Unknown zip code	7463	215 (2.9)
First (0% to <3.8%)	707 524	11 648 (1.7)
Second (3.8% to <7.4%)	2 302 844	41 882 (1.8)
Third (7.4% to <12.3%)	2 413 475	51 757 (2.1)
Fourth (≥12.3%)	1 638 305	43 090 (2.6)
Quartile of Elixhauser Comorbidity Index score^e^		
First (≤−2)	1 705 326	51 616 (3.0)
Second (−1 to 4)	1 817 314	31 683 (1.7)
Third (5 to 16)	1 710 545	33 202 (1.9)
Fourth (≥17)	1 836 426	32 091 (1.8)

^a^ Data were taken from the 2021-2022 Medicare Provider Analysis and Review file,^27^ 2021-2022 Master Beneficiary Summary File,^28^ and the 2019 American Community Survey.^29^

^b^ Race and ethnicity obtained from the Research Triangle Institute race code, which uses an algorithm to identify additional Asian and Hispanic beneficiaries. The other race category was aggregated due to small samples and including Asian or Pacific Islander, American Indian or Alaska Native, other race, and unknown race.

^c^ End-stage kidney disease category includes beneficiaries with both disability insurance benefits and end-stage kidney disease.

^d^ Quartiles of zip code–level poverty rate are among all US zip code tabulation areas. Zip code–level poverty rate is measured among residents 65 years and older.

^e^ Quartiles of Elixhauser Comorbidity Index score are among the sample population. The Elixhauser Comorbidity Index range includes negative scores due to certain comorbidities being negatively correlated with in-hospital mortality.

### Association of Z Code Prevalence With Medical Complexity

Within-hospital Z code prevalence was higher for beneficiaries with the lowest vs highest Elixhauser Comorbidity Index scores (2.8% vs 1.5%; Table 2). This pattern was evident among dual-eligible beneficiaries (4.8% vs 1.6%) and non–dual-eligible beneficiaries (2.2% vs 1.5%), as well as among beneficiaries living in zip codes in the highest quartile of poverty (3.6% vs 1.6%) and in zip codes in the lowest quartile (2.3% vs 1.6%).

**Table 2.** Z Code Prevalence Across Levels of Medical Complexity Among Medicare Beneficiary Hospital Inpatients^a^^,^^b^

Medical complexity measure	Medicare beneficiaries’ first hospitalization in January to November 2022, %				
	Full sample	Dual eligibility status^c^		Zip code poverty quartile^d^	
		Dual eligible	Not dual eligible	Highest	Lowest
Quartile of No. of diagnoses^e^					
First (1 to 11)	1.4	2.7	1.2	1.9	1.2
Second (12 to 16)	1.9	3.1	1.7	2.4	1.7
Third (17 to 22)	2.4	3.4	2.2	2.9	2.3
Fourth (23 to 25)	2.2	2.3	2.1	2.4	2.1
Quartile of Elixhauser Comorbidity Index score^e^					
First (≤−2)	2.8	4.8	2.2	3.6	2.3
Second (−1 to 4)	1.6	2.5	1.5	1.9	1.5
Third (5 to 16)	1.7	2.2	1.6	2.0	1.7
Fourth (≥17)	1.5	1.6	1.5	1.6	1.6
Diagnosis Related Group code type					
Primary mental health and/or disability	19.2	22.9	16.6	21.0	16.5
Primary general medical	1.6	2.2	1.5	1.9	1.5
Primary surgical	0.7	0.9	0.8	0.8	0.8

^a^ Prevalence of Z codes estimated from marginal means after fitting admission-level linear regression models for presence of a Z code as a function of each medical complexity measure and hospital fixed effects. Separate models were evaluated for each population listed in the columns.

^b^ Data were taken from the 2021-2022 Medicare Provider Analysis and Review file,^27^ 2021-2022 Master Beneficiary Summary File,^28^ and the 2019 American Community Survey.^29^

^c^ Dual eligible status was identified as patients with 12 months of full dual eligibility preceding admission.

^d^ Quartiles of zip code–level poverty rate are among all US zip code tabulation areas. Zip code–level poverty rate is measured among residents 65 years and older.

^e^ Quartiles of number of diagnoses and Elixhauser Comorbidity Index were calculated in the full sample. The Elixhauser Comorbidity Index range includes negative scores due to certain comorbidities being negatively correlated with in-hospital mortality.

The association between total number of diagnoses and Z code prevalence was more heterogeneous. Among dual-eligible beneficiaries, within-hospital Z code prevalence was higher among patients in the third vs first quartile of reported diagnoses (3.4% vs 2.7%) and lowest among patients with the most reported diagnoses (2.3%). Among patients living in zip codes in the highest quartile of poverty, Z code prevalence was highest in the third quartile of reported diagnoses (2.9%) relative to other quartiles (first, 1.9%; second, 2.4%; fourth, 2.4%).

Overall, within-hospital Z code prevalence was substantially higher among beneficiaries admitted with a primary mental health and/or disability DRG code (19.2%) than those with primary general medical (1.6%) or surgical (0.7%) codes. This pattern was present across all subgroups.

### Association of Z Code Prevalence With Mortality Risk

**Figure 1.**
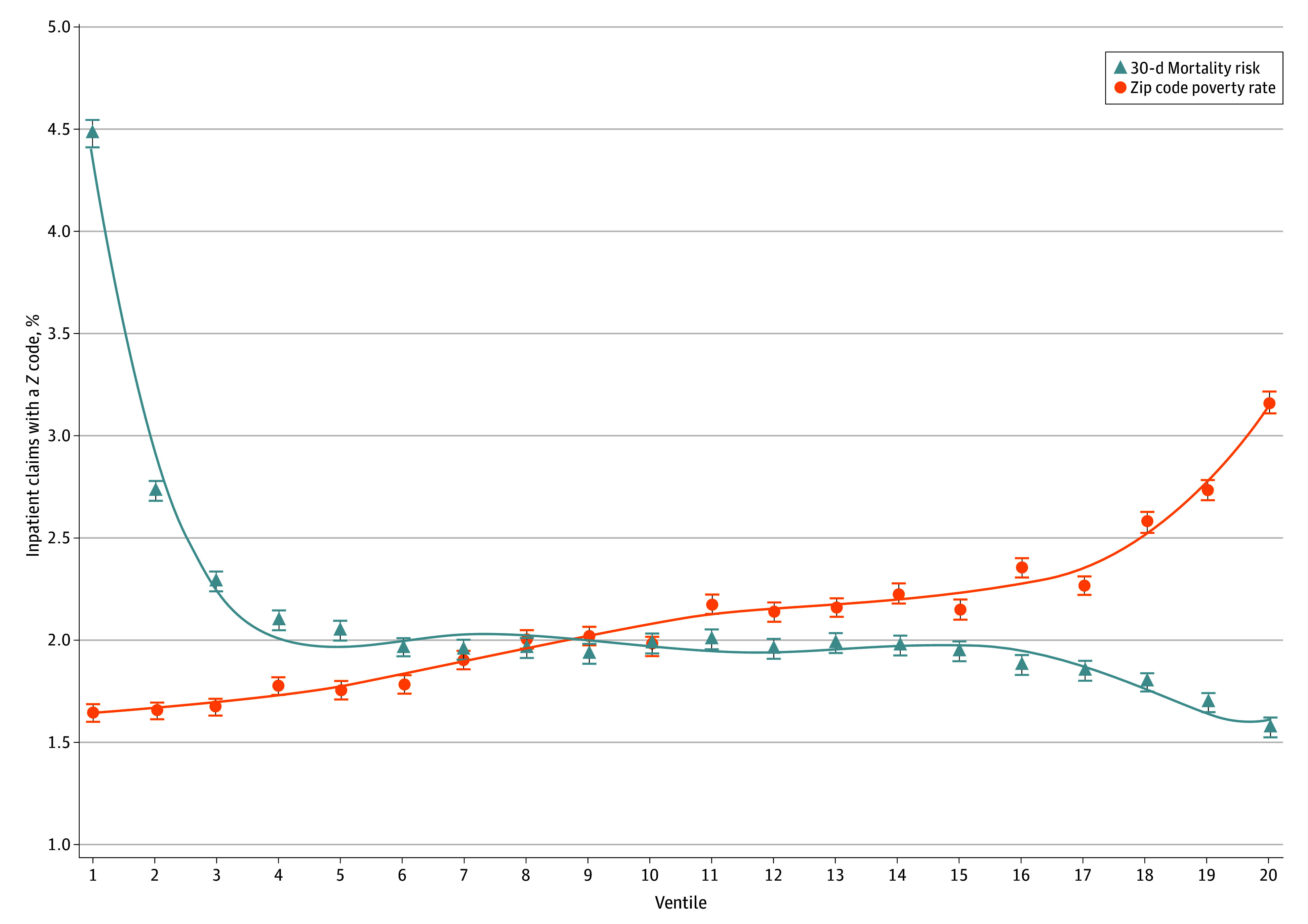
Inpatient Z Coding Rate by Mortality Risk and Area-Level Poverty This plot shows the percentage of inpatient hospital claims with a Z code across ventiles of 30-day predicted mortality risk and ventiles of zip code poverty rate. Predicted mortality risk is modeled from a linear regression of 30-day mortality based on age, sex, original Medicare entitlement reason, 38 Elixhauser Comorbidity Index comorbidities, Diagnosis Related Group code, and hospital fixed effects. Each beneficiary is assigned a predicted mortality risk score based on individual-level and admission-level characteristics from the regression model, with hospital effects excluded. Zip code poverty rates are 5-year averages among residents 65 years and older. Data were taken from the 2021-2022 Medicare Provider Analysis and Review file,^27^ 2021-2022 Master Beneficiary Summary File,^28^ and the 2019 American Community Survey.^29^ Error bars indicate 95% CIs.

Z code prevalence was negatively correlated with predicted 30-day postdischarge mortality risk (Figure 1). The within-hospital prevalence of Z codes declined most substantially between the first and fourth ventiles of predicted mortality risk. As expected, Z code prevalence was positively correlated with zip code–level poverty.

**Figure 2.**
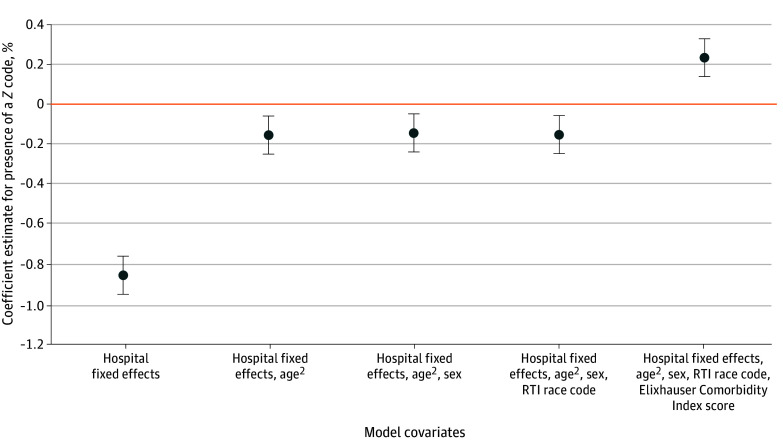
Regression Estimates for Association Between the Presence of a Z Code and 30-Day Postdischarge Mortality, With Covariates Added Sequentially This plot shows the coefficient estimate of presence of a Z code in a linear regression model (independent variable) for the outcome of postdischarge 30-day mortality, with covariates sequentially added to the model. The original model includes hospital fixed effects; the square of age, sex, race and ethnicity, and Elixhauser Comorbidity Index were added sequentially to the following models. Race and ethnicity were obtained from the Research Triangle Institute (RTI) race code, which uses an algorithm to identify additional Asian and Hispanic beneficiaries. Data were taken from the 2021-2022 Medicare Provider Analysis and Review file,^27^ 2021-2022 Master Beneficiary Summary File,^28^ and the 2019 American Community Survey.^29^ Error bars indicate 95% CIs.

The presence of a Z code was associated with a lower probability of 30-day mortality (5.1% vs 4.2%; difference, −0.9 percentage points; 95% CI, −1.0 to −0.8) when adjusting for hospital fixed effects but no other covariates (Figure 2). The addition of the square of age attenuated this association (5.0% vs 4.8%; difference, −0.2 percentage points; 95% CI, −0.2 to 0.1). Further addition of sex (5.0% vs 4.9%; difference, −0.1 percentage points; 95% CI, −0.2 to 0), race and ethnicity (5.1% vs 4.9%; difference, −0.2 percentage points; 95% CI, −0.2 to −0.1), and Elixhauser Comorbidity Index score (5.2% vs 5.4%; difference, 0.2 percentage points; 95% CI, 0.1 to 0.3) yielded similar estimates of mortality differences associated with Z code documentation.

### Supplementary Analyses

Z code prevalence was higher among patients with at least 2 hospitalizations in the year before the index hospitalization (2.6%) than patients with 1 or 0 prior hospitalizations (2.1% and 1.8%, respectively; eTable 2 in Supplement 1). This was consistent across strata of dual eligibility status and residence in zip codes in the highest and lowest quartiles of poverty. The negative correlation between Z code prevalence and 30-day mortality risk was most pronounced among patients with mental health and/or disability-related admissions (eFigure 1 in Supplement 1). For this population, Z code prevalence was highest among patients in the third ventile of 30-day mortality risk (31.0%) and lowest among patients in the highest ventile (5.2%). For patients with general medical admissions, Z code prevalence was 3.5% in the lowest ventile of mortality risk and 1.2% in the highest ventile (eFigure 2 in Supplement 1). For surgical admissions, Z code prevalence was low overall and increased modestly with mortality risk (eFigure 3 in Supplement 1). Z code prevalence across ventiles of 30-day mortality risk and area-level poverty were similar among fee-for-service Medicare vs Medicare Advantage beneficiaries (eFigures 4 and 5 in Supplement 1).

We observed a weak positive correlation (ρ = 0.22) between hospital-level Z code prevalence and the mean number of diagnoses per admission (eFigure 6 in Supplement 1). We found a consistent association between increasing Elixhauser Comorbidity Index levels and lower Z code prevalence across tertiles of hospital diagnostic coding intensity (eTable 3 in Supplement 1). Results were consistent in analyses that used the beneficiary’s last admission from January to November 2022 (eTable 4 in Supplement 1).

## Discussion

In this national study of hospitalized Medicare beneficiaries, we found that documentation of Z codes was uncommon, variable, and susceptible to measurement bias. Z codes were less commonly documented among patients with higher estimated risks of 30-day mortality. These patterns persisted for dual-eligible and non–dual-eligible beneficiaries and residents of high-poverty and low-poverty zip codes.

Our results illustrate the different pathways by which measurement bias manifests. Documentation of Z codes was related to patients’ prior health care use; patients who had repeated health care interactions were more likely to have a Z code. If documentation of social risk is contingent on utilization, then organizations may be less likely to detect social risk among patients with barriers to accessing care.^12^ Conditional on receiving care, documentation of social risk varied by clinical complexity; Z codes were reported less often among more clinically complex patients, as measured by Elixhauser Comorbidity Index risk scores and predicted 30-day postdischarge mortality. Even when we focused on patients admitted for mental health conditions—who tend to be younger, have fewer comorbidities, and, thus, lower estimated risks for mortality—Z code documentation remained less common among patients with higher predicted mortality risk. These patterns suggest measurement bias in the use of Z codes rather than true underlying differences in social risk, especially given abundant evidence that social risk factors and poor health are positively correlated. These 2 mechanisms of documentation bias—namely, dependence on utilization to document social risk and biased risk detection by clinical complexity—illustrate how measurement biases can distort data collection intended to advance population health.

An implication of these measurement biases is that Z codes may undercount individuals with concomitant social and clinical risk factors, both of which are linked to adverse outcomes. We found that the presence of a Z code was negatively correlated with observed mortality before adjusting for clinical risk, despite the well-established positive correlation between health and social risks.^8,9,10,11^ A spurious negative correlation between Z codes and mortality could misleadingly suggest that disadvantaged individuals face lower risks of adverse outcomes and, thus, have fewer resource needs than their social risk factors warrant. As value-based payment approaches increasingly consider social risk, our results indicate that existing patterns of Z code documentation could perpetuate resource misallocation.

Relying on health care organizations to accurately capture social risk is difficult for several reasons. First, disadvantaged patients experience barriers to care^30,31,32^ such that relying on utilization to measure social risk will introduce selection bias. Similar biases have been found with risk prediction models, which underestimate future health care needs in groups that experience difficulty obtaining care.^12^ Second, relying on utilization and billing data to document social risks may exacerbate differences in how organizations monitor and address health-related social needs, since larger, well-resourced organizations may be better able to capture social risk due to workforce capacity and existing resources for billing and coding.^33^ Safety-net hospitals, which disproportionately serve low-income patients and provide uncompensated care, may be less able to capitalize on Z coding efforts due to financial challenges and staffing shortages.^34,35,36^ Although technological advances in electronic health records may improve documentation in resource-limited settings in the future, dedicating additional resources toward coding may divert attention and funding from broader population health goals. Prioritizing investment in sectors such as education and employment could be more beneficial in addressing social needs than additional investments in health care delivery.^37^

Understanding social risk is important because clinicians who understand the social context can better deliver patient-centered care. Instead of bolstering billing capacity to capture social risk, hospitals could incorporate existing, validated, and often freely available population-level data on social risk into electronic health records. Although area-level measures do not perfectly map onto individual social risk, several measures are well correlated in urban areas.^38^ Health care organizations could also consider partnering with public health agencies that are simultaneously engaging in efforts to screen and detect social risk.^39^ If health care organizations continue to be responsible for capturing social risk, then policymakers should consider patients’ clinical severity when determining the accuracy of such capture.

### Limitations

This study has limitations. First, we focused only on Z codes recording during hospitalization. Outpatient patterns of Z code use may differ due to the longitudinal nature of care. Second, we relied on area-level measures of socioeconomic disadvantage, which only partially correlate with individual-level disadvantage. Third, our sample was limited to 2022, and Z code use may have increased since this period. Finally, there may be residual confounding by hospital coding intensity, in which hospitals that code intensively may select for healthier patients, contributing to a spurious association between clinical severity and social risk. We sought to address this with hospital fixed effects. We also tested whether the negative relationship between Elixhauser Comorbidity Index score and likelihood of Z code documentation held across hospitals with varying coding intensities and found that the negative association persisted across all tertiles of hospital coding intensity. Still, there may be unobserved factors influencing both the documentation of medical diagnoses and Z codes.

## Conclusions

In this retrospective cohort study, Z codes, which were designed to capture social risk in the medical record, were susceptible to measurement bias. Z code use patterns likely underrepresented social risk among clinically complex patients, resulting in a spurious negative association between social risk and mortality. These patterns have implications for risk adjustment, value-based payment, and resource allocation. Health care organizations might consider using external, population-level data sources rather than Z codes to measure social risk.

## Notes

Supplement 1. **eTable 1.** Description and Sample Prevalence of Z-Codes **eTable 2.** Within-Hospital Z-Coding Prevalence Across Levels of Prior-Year Hospital Utilization Among Medicare Beneficiary Hospital Inpatients in 2022 **eTable 3.** Z-Coding Rates Across Levels of Medical Complexity Among Medicare Beneficiary Hospital Inpatients, by Dual Status and Area-Level Poverty **eTable 4.** Sensitivity Analysis With Medicare Beneficiaries' Last Hospitalization in January to November 2022 **eFigure 1.** Prevalence of Z-Coding Among Mental Health Inpatients in 2022 Across Ventiles of Mortality Risk and Area-Level Poverty **eFigure 2.** Prevalence of Z-Coding Among General Medical Inpatients in 2022 Across Ventiles of Mortality Risk and Area-Level Poverty **eFigure 3.** Prevalence of Z-Coding Among Surgical Inpatients in 2022 Across Ventiles of Mortality Risk and Area-Level Poverty **eFigure 4.** Prevalence of Z-Coding Among Fee-for-Service Medicare Inpatients in 2022 Across Ventiles of Mortality Risk and Area-Level Poverty **eFigure 5.** Prevalence of Z-Coding Among Medicare Advantage Inpatients in 2022 Across Ventiles of Mortality Risk and Area-Level Poverty **eFigure 6.** Scatterplot of Hospital-Level Z-Coded Admission Rate vs Mean Number of Diagnoses Per Admission

Supplement 2. Data Sharing Statement
