# Supplementary material for: Measurement Bias in Documentation of Social Risk Among Medicare Beneficiaries
Source: JAMA Health Forum. 2025 Jul 18;6(7):e251923. doi: 10.1001/jamahealthforum.2025.1923 (PMC12274977; doi:10.1001/jamahealthforum.2025.1923)
Supplement: Supplement 1. — eTable 1. Description and Sample Prevalence of Z-Codes eTable 2. Within-Hospital Z-Coding Prevalence Across Levels of Prior-Year Hospital Utilization Among Medicare Beneficiary Hospital Inpatients in 2022 eTable 3. Z-Coding Rates Across Levels of Medical Complexity Among Medicare Beneficiary Hospital Inpatients, by Dual Status and Area-Level Poverty eTable 4. Sensitivity Analysis With Medicare Beneficiaries' Last Hospitalization in January to November 2022 eFigure 1. Prevalence of Z-Coding Among Mental Health Inpatients in 2022 Across Ventiles of Mortality Risk and Area-Level Poverty eFigure 2. Prevalence of Z-Coding Among General Medical Inpatients in 2022 Across Ventiles of Mortality Risk and Area-Level Poverty eFigure 3. Prevalence of Z-Coding Among Surgical Inpatients in 2022 Across Ventiles of Mortality Risk and Area-Level Poverty eFigure 4. Prevalence of Z-Coding Among Fee-for-Service Medicare Inpatients in 2022 Across Ventiles of Mortality Risk and Area-Level Poverty eFigure 5. Prevalence of Z-Coding Among Medicare Advantage Inpatients in 2022 Across Ventiles of Mortality Risk and Area-Level Poverty eFigure 6. Scatterplot of Hospital-Level Z-Coded Admission Rate vs Mean Number of Diagnoses Per Admission [file jamahealthforum-e251923-s001.pdf]

Supplemental Online Content

Chatterjee P, Macneal E, Roberts ET. Measurement bias in documentation of social risk among Medicare beneficiaries. *JAMA Health Forum.* 2025;6(7):e251923. doi:10.1001/jamahealthforum.2025.1923

eTable 1. Description and Sample Prevalence of Z-Codes
eTable 2. Within-Hospital Z-Coding Prevalence Across Levels of Prior-Year Hospital Utilization Among Medicare Beneficiary Hospital Inpatients in 2022
eTable 3. Z-Coding Rates Across Levels of Medical Complexity Among Medicare Beneficiary Hospital Inpatients, by Dual Status and Area-Level Poverty
eTable 4. Sensitivity Analysis With Medicare Beneficiaries' Last Hospitalization in January to November 2022
eFigure 1. Prevalence of Z-Coding Among Mental Health Inpatients in 2022 Across Ventiles of Mortality Risk and Area-Level Poverty
eFigure 2. Prevalence of Z-Coding Among General Medical Inpatients in 2022 Across Ventiles of Mortality Risk and Area-Level Poverty
eFigure 3. Prevalence of Z-Coding Among Surgical Inpatients in 2022 Across Ventiles of Mortality Risk and Area-Level Poverty
eFigure 4. Prevalence of Z-Coding Among Fee-for-Service Medicare Inpatients in 2022 Across Ventiles of Mortality Risk and Area-Level Poverty
eFigure 5. Prevalence of Z-Coding Among Medicare Advantage Inpatients in 2022 Across Ventiles of Mortality Risk and Area-Level Poverty
eFigure 6. Scatterplot of Hospital-Level Z-Coded Admission Rate vs Mean Number of Diagnoses Per Admission

This supplementary material has been provided by the authors to give readers additional information about their work.

© 2025 Chatterjee P et al. *JAMA Health Forum*.

eTable 1. Description and Sample Prevalence of Z-Codes

Population: Medicare beneficiaries' first hospitalization in 2022

Code	Description	Proportion of sample with specific Z-code
Z55	Problems related to education and literacy	0.01%
Z56	Problems related to employment and unemployment	0.17%
Z57	Occupational exposure to risk factors	0.02%
Z58	Problems related to physical environment	0.00%
Z59	Problems related to housing and economic circumstances	0.76%
Z60	Problems related to social environment	0.66%
Z62	Problems related to upbringing	0.06%
Z63	Other problems related to primary support group, including family circumstances	0.53%
Z64	Problems related to certain psychosocial circumstance	0.00%
Z65	Problems related to other psychosocial circumstances	0.05%

© 2025 Chatterjee P et al. *JAMA Health Forum*.

eTable 2. Within-Hospital Z-Coding Prevalence Across Levels of Prior-Year Hospital Utilization Among Medicare Beneficiary Hospital Inpatients in 2022

Population: Medicare beneficiaries' first hospitalization in 2022

Number of hospitalizations in year prior to indexed hospitalization	**Full sample**	**Dual eligibles**	**Non-dual eligibles**	**Patients living in zip codes in the top quartile of poverty**	**Patients living in zip codes in the bottom quartile of poverty**
0	1.8%	2.6%	1.6%	2.1%	1.7%
1	2.1%	3.0%	1.8%	2.5%	1.8%
>=2	2.6%	3.8%	2.1%	3.3%	2.1%

Notes: Reported rates of Z-coding are marginal means from linear regression models of Z-coding based on number of prior-year hospitalizations and hospital fixed effects. Separate models are evaluated for each population listed in the columns. Dual eligibles are identified as patients with 12 months of full dual eligibility preceding admission. Quartiles of zip code-level poverty rate are among all US zip code tabulation areas. Zip code-level poverty rate is measured among residents aged >=65.

Source: MedPAR 2021-2022, MBSF 2021-2022, ACS 2019

© 2025 Chatterjee P et al. *JAMA Health Forum*.

eFigure 1. Prevalence of Z-Coding Among Mental Health Inpatients in 2022 Across Ventiles of Mortality Risk and Area-Level Poverty

Population: Medicare beneficiaries whose first hospitalization in January-November 2022 was a mental health admission

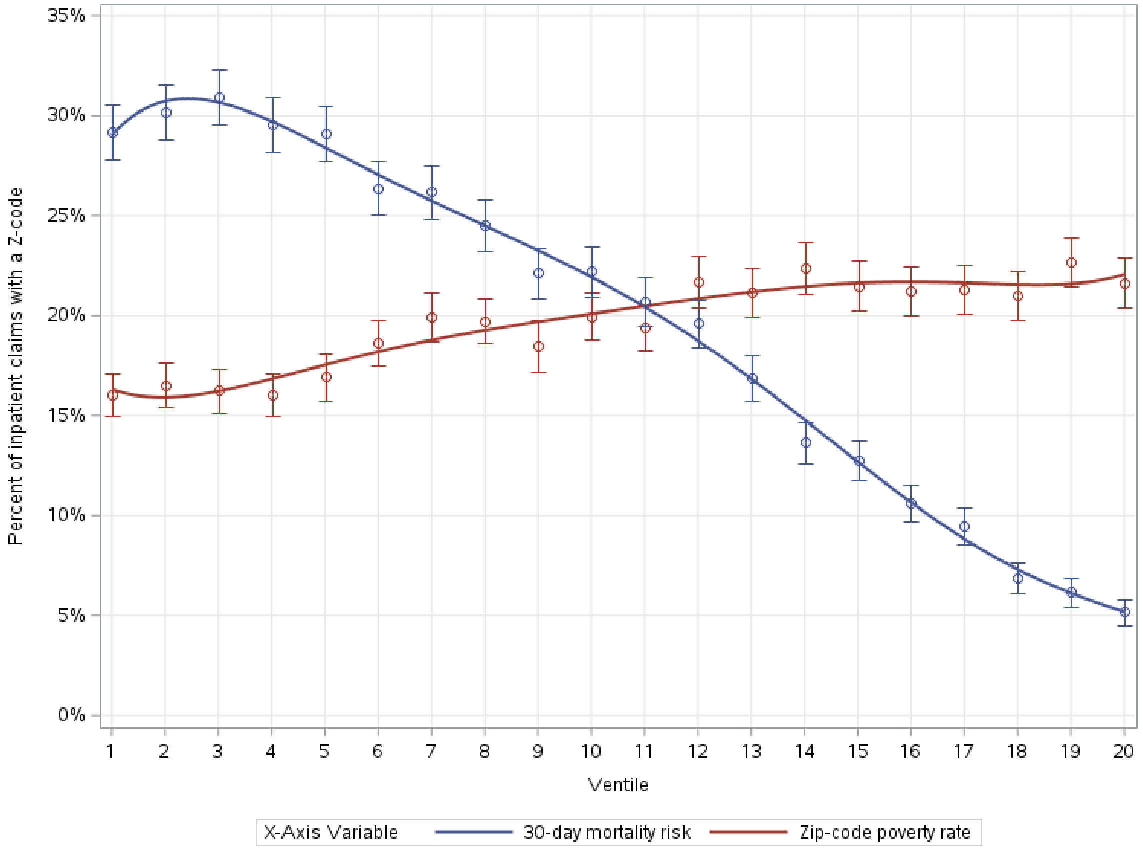

Notes: This plot shows the proportion of inpatient hospital claims with a Z-code across ventiles of 30-day predicted mortality risk and ventiles of zip code poverty rate. Predicted mortality risk is modeled from a linear regression of 30-day mortality based on the square of age, sex, original Medicare entitlement reason, 38 Elixhauser comorbidities, DRG code, and hospital fixed effects. Each beneficiary is a assigned a predicted mortality risk score based on individual- and admission-level characteristics from the regression model, with hospital effects excluded. Zip code poverty rates are 5-year averages among residents aged 65 and older.

Source: MedPAR 2021-2022, MBSF 2021-2022, ACS 2019

© 2025 Chatterjee P et al. *JAMA Health Forum*.

eFigure 2. Prevalence of Z-Coding Among General Medical Inpatients in 2022 Across Ventiles of Mortality Risk and Area-Level Poverty

Population: Medicare beneficiaries whose first hospitalization in January-November 2022 was a general medical admission

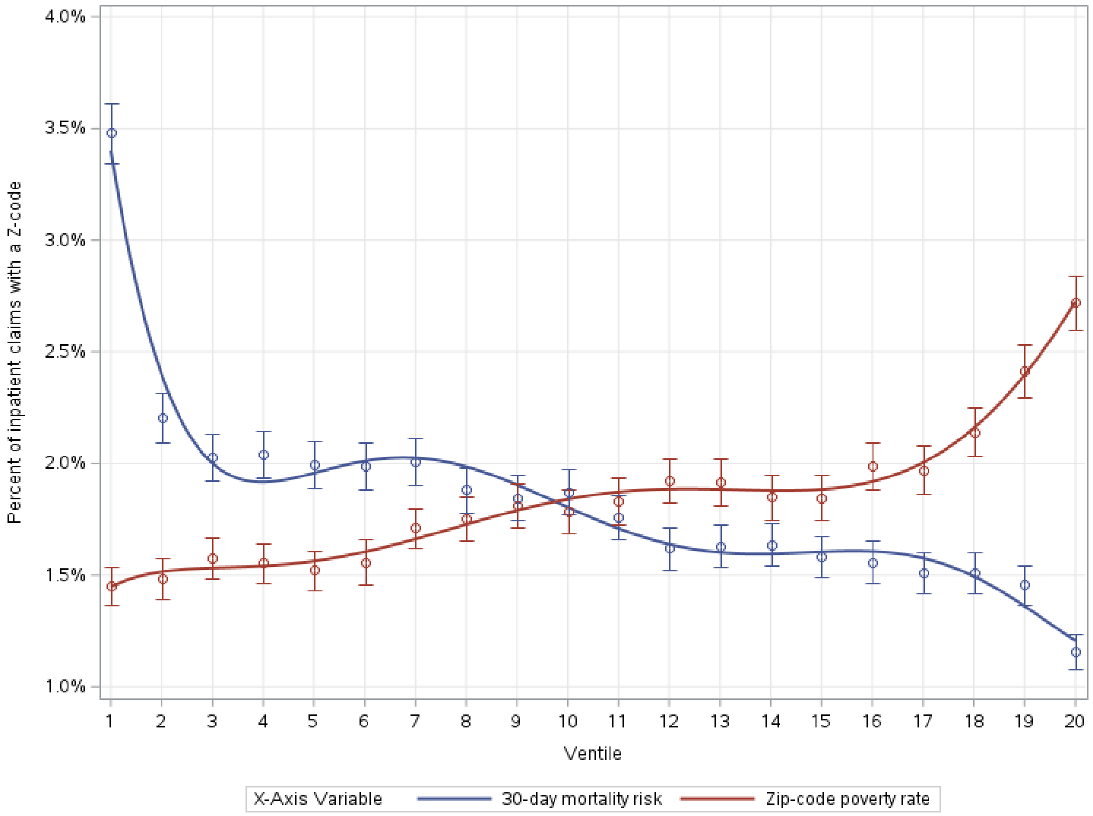

Notes: This plot shows the proportion of inpatient hospital claims with a Z-code across ventiles of 30-day predicted mortality risk and ventiles of zip code poverty rate. Predicted mortality risk is modeled from a linear regression of 30-day mortality based on the square of age, sex, original Medicare entitlement reason, 38 Elixhauser comorbidities, DRG code, and hospital fixed effects. Each beneficiary is a assigned a predicted mortality risk score based on individual- and admission-level characteristics from the regression model, with hospital effects excluded. Zip code poverty rates are 5-year averages among residents aged 65 and older.

Source: MedPAR 2021-2022, MBSF 2021-2022, ACS 2019

© 2025 Chatterjee P et al. *JAMA Health Forum*.

eFigure 3. Prevalence of Z-Coding Among Surgical Inpatients in 2022 Across Ventiles of Mortality Risk and Area-Level Poverty

Population: Medicare beneficiaries whose first hospitalization in January-November 2022 was a surgical admission

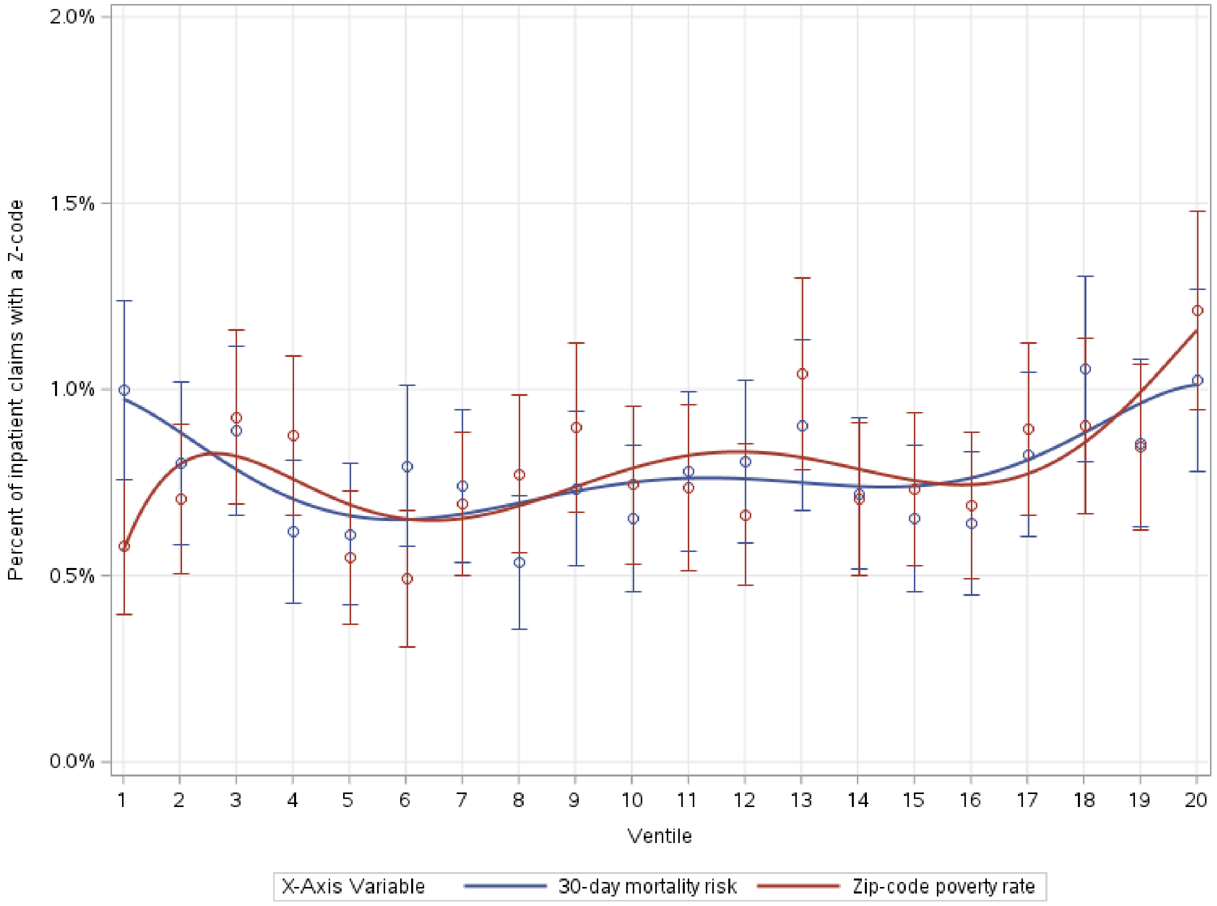

Notes: This plot shows the proportion of inpatient hospital claims with a Z-code across ventiles of 30-day predicted mortality risk and ventiles of zip code poverty rate. Predicted mortality risk is modeled from a linear regression of 30-day mortality based on the square of age, sex, original Medicare entitlement reason, 38 Elixhauser comorbidities, DRG code, and hospital fixed effects. Each beneficiary is a assigned a predicted mortality risk score based on individual- and admission-level characteristics from the regression model, with hospital effects excluded. Zip code poverty rates are 5-year averages among residents aged 65 and older.

Source: MedPAR 2021-2022, MBSF 2021-2022, ACS 2019

© 2025 Chatterjee P et al. *JAMA Health Forum*.

eFigure 4. Prevalence of Z-Coding Among Fee-for-Service Medicare Inpatients in 2022 Across Ventiles of Mortality Risk and Area-Level Poverty

Population: Fee-for-service Medicare beneficiaries' first hospitalization in January-November 2022

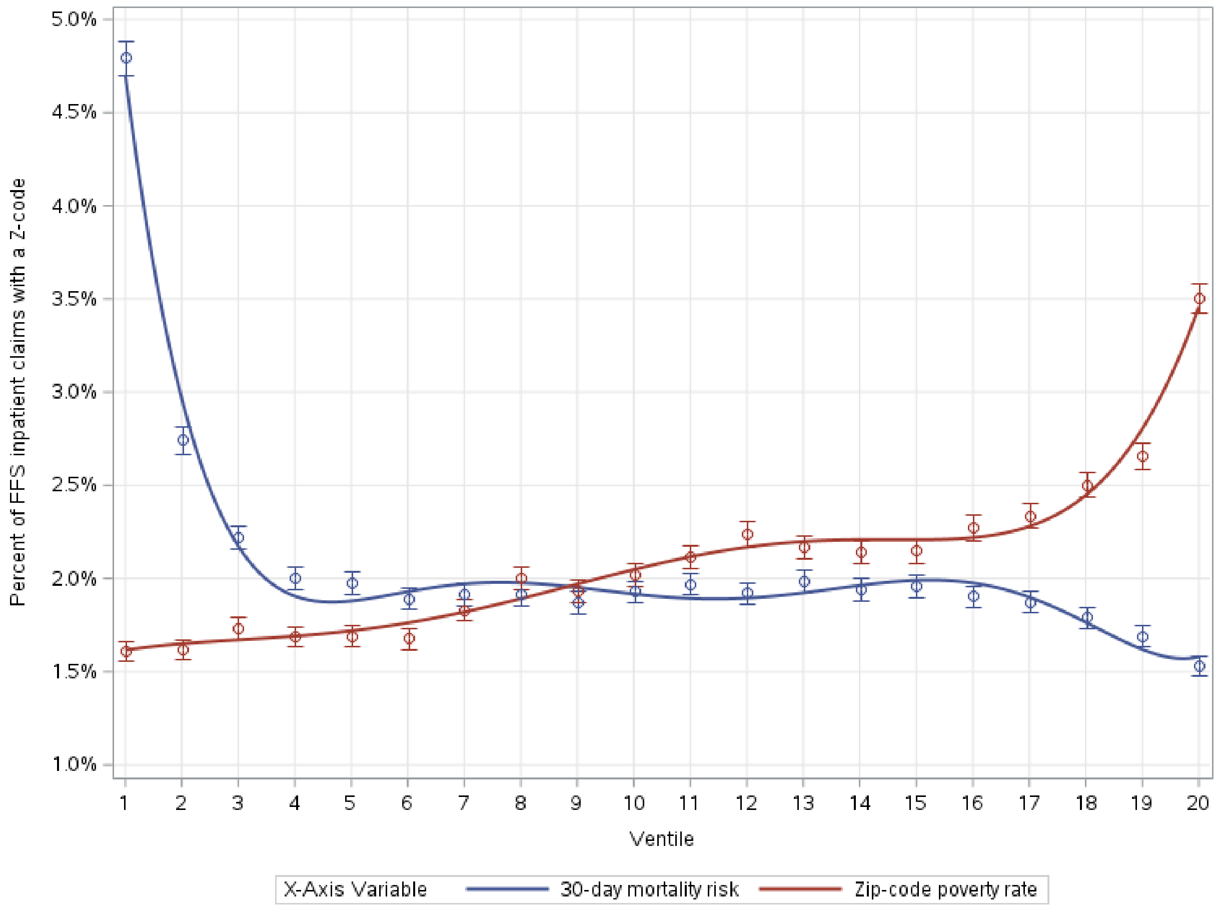

Notes: This plot shows the proportion of inpatient hospital claims with a Z-code across ventiles of 30-day predicted mortality risk and ventiles of zip code poverty rate. Predicted mortality risk is modeled from a linear regression of 30-day mortality based on the square of age, sex, original Medicare entitlement reason, 38 Elixhauser comorbidities, DRG code, and hospital fixed effects. Each beneficiary is a assigned a predicted mortality risk score based on individual- and admission-level characteristics from the regression model, with hospital effects excluded. Zip code poverty rates are 5-year averages among residents aged 65 and older.

Source: MedPAR 2021-2022, MBSF 2021-2022, ACS 2019

© 2025 Chatterjee P et al. *JAMA Health Forum*.

eFigure 5. Prevalence of Z-Coding Among Medicare Advantage Inpatients in 2022 Across Ventiles of Mortality Risk and Area-Level Poverty

Population: Medicare Advantage beneficiaries' first hospitalization in January-November 2022

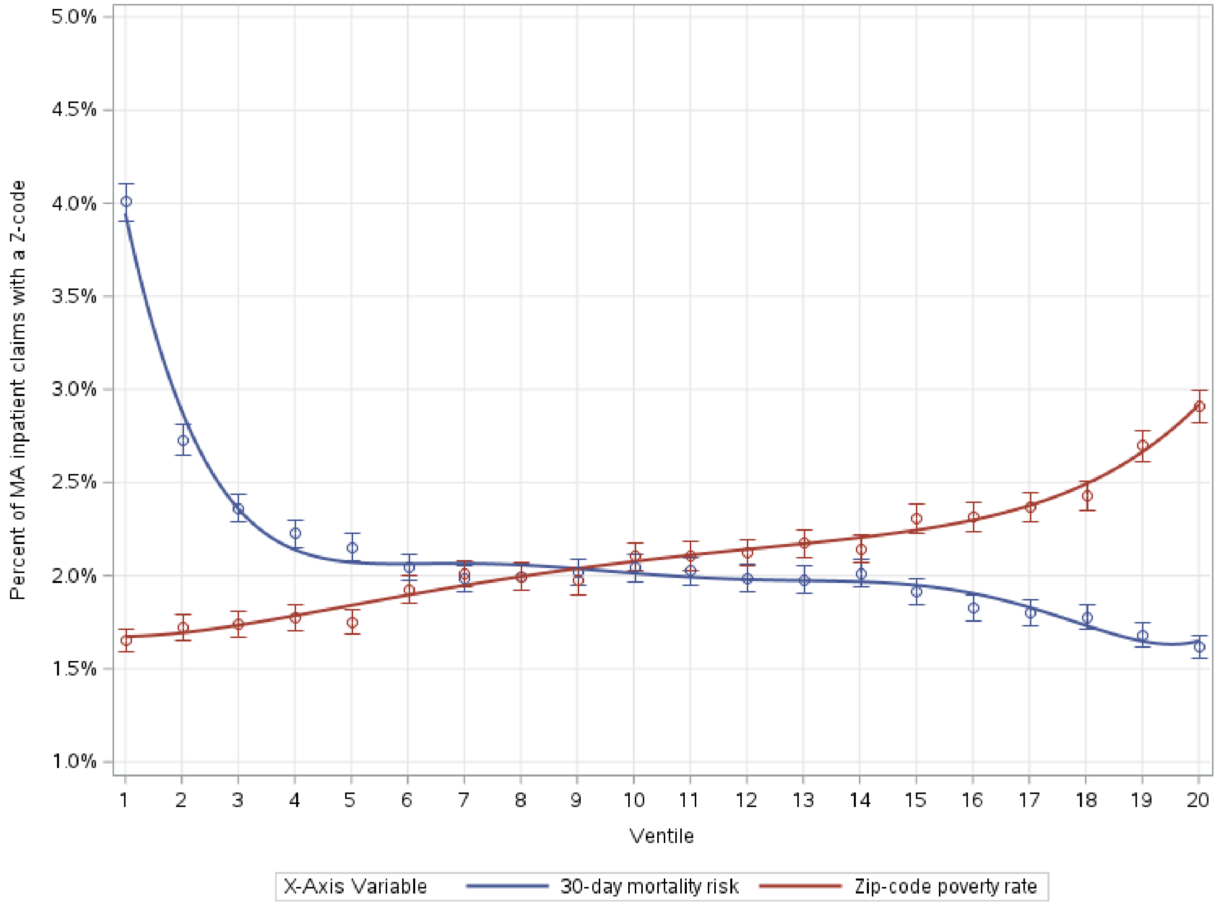

Notes: This plot shows the proportion of inpatient hospital claims with a Z-code across ventiles of 30-day predicted mortality risk and ventiles of zip code poverty rate. Predicted mortality risk is modeled from a linear regression of 30-day mortality based on the square of age, sex, original Medicare entitlement reason, 38 Elixhauser comorbidities, DRG code, and hospital fixed effects. Each beneficiary is a assigned a predicted mortality risk score based on individual- and admission-level characteristics from the regression model, with hospital effects excluded. Zip code poverty rates are 5-year averages among residents aged 65 and older.

Source: MedPAR 2021-2022, MBSF 2021-2022, ACS 2019

© 2025 Chatterjee P et al. *JAMA Health Forum*.

eFigure 6. Scatterplot of Hospital-Level Z-Coded Admission Rate vs Mean Number of Diagnoses Per Admission

Population: General and critical access hospitals with Medicare inpatient admissions in 2022

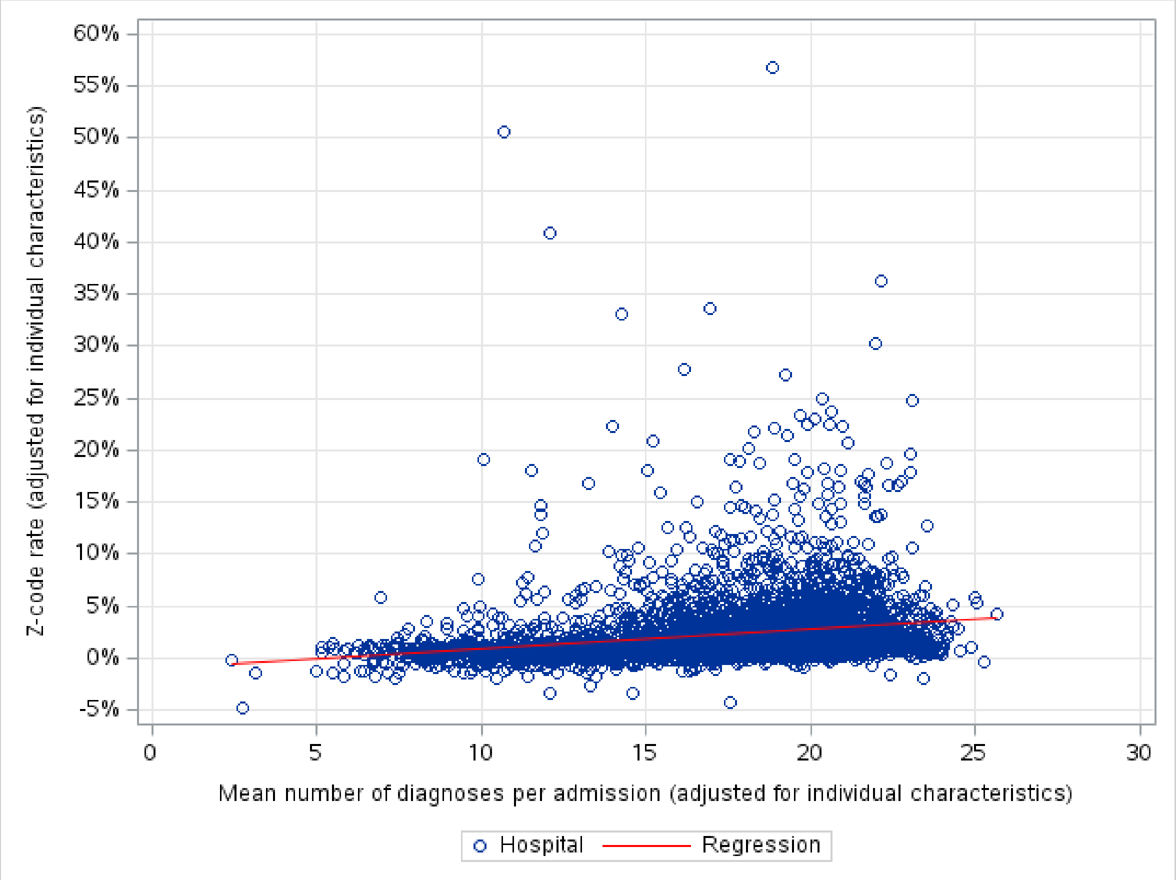

Rho: 0.22

Notes: Adjusted estimates of Z-coding rate and mean number of diagnoses per admission are calculated from admission-level linear regression models of presence of a Z-code and number of diagnosis codes, respectively, based on patient characteristics (square of age, disability, dual-eligibility status, and residence in a high-poverty ZIP code) and hospital fixed effects. Hospital-level estimates reflect hospital effect coefficients centered at the grand means of Z-coding prevalence and number of diagnoses.

Source: MedPAR 2021-2022, MBSF 2021-2022, ACS 2019

© 2025 Chatterjee P et al. *JAMA Health Forum*.

eTable 3. Z-Coding Rates Across Levels of Medical Complexity Among Medicare Beneficiary Hospital Inpatients, by Dual Status and Area-Level Poverty

Population: Medicare beneficiaries' first hospitalization in January-November 2022

**Medical complexity measure**	**Hospitals in lowest tertile of coding intensity**	**Hospitals in middle tertile of coding intensity**	**Hospitals in highest tertile of coding intensity**
Number of diagnoses			
Q1 (1-11)	0.9%	1.5%	1.8%
Q2 (12-16)	1.2%	2.1%	2.4%
Q3 (17-22)	1.6%	2.6%	2.9%
Q4 (23-25)	1.4%	2.4%	2.6%
Elixhauser In-Hospital Mortality Index			
Q1 (<=-2)	1.7%	3.0%	3.5%
Q2 (-1 to 4)	0.8%	1.8%	2.3%
Q3 (5 to 16)	0.9%	2.0%	2.4%
Q4 (>=17)	0.7%	1.8%	2.1%
DRG code type			
Primary mental health and/or disability	9.8%	18.5%	22.7%
Primary general medical	0.8%	1.9%	2.3%
Primary surgical	0.4%	0.8%	1.4%

Notes: Tertiles of coding intensity are based on 100% of 2022 admissions in the sample hospitals. Prevalence of Z-coding is derived from marginal means from linear regression models of Z-coding based on each medical complexity measure and hospital fixed effects. Separate models are evaluated for each population listed in the columns. Quartiles of number of diagnoses and Elixhauser In-Hospital Mortality Index are among the full sample population. The Elixhauser In-Hospital Mortality Index range includes negative scores due to certain comorbidities being negatively correlated with in-hospital mortality. Quartiles of zip code-level poverty rate are among all US zip code tabulation areas. Zip code-level poverty rate is measured among residents aged >=65.

Source: MedPAR 2021-2022, MBSF 2021-2022, ACS 2019

© 2025 Chatterjee P et al. *JAMA Health Forum*.

eTable 4. Sensitivity Analysis With Medicare Beneficiaries' Last Hospitalization in January to November 2022

Population: Medicare beneficiaries' last hospitalization in January-November 2022

**Medical complexity measure**	**Full sample**	**Dual eligibles**	**Non-dual eligibles**	**Patients living in zip codes in the top quartile of poverty**	**Patients living in zip codes in the bottom quartile of poverty**
Number of diagnoses					
Q1 (1-11)	1.4%	2.9%	1.1%	1.9%	1.3%
Q2 (12-16)	2.0%	3.4%	1.6%	2.5%	1.8%
Q3 (17-22)	2.5%	3.8%	2.1%	3.0%	2.3%
Q4 (23-25)	2.2%	2.7%	2.0%	2.4%	2.2%
Elixhauser In-Hospital Mortality Index					
Q1 (<=-2)	2.9%	5.3%	2.1%	3.8%	2.4%
Q2 (-1 to 4)	1.6%	2.8%	1.4%	2.0%	1.6%
Q3 (5 to 16)	1.8%	2.5%	1.6%	2.1%	1.8%
Q4 (>=17)	1.5%	1.9%	1.4%	1.7%	1.6%
DRG code type					
Primary mental health and/or disability	19.7%	23.7%	16.0%	21.6%	16.9%
Primary general medical	1.7%	2.5%	1.4%	2.0%	1.5%
Primary surgical	0.7%	0.9%	0.8%	0.8%	0.9%

Notes: Prevalence of Z-coding is derived from marginal means from linear regression models of Z-coding based on each medical complexity measure and hospital fixed effects. Separate models are evaluated for each population listed in the columns. Dual eligibles are identified as patients with 12 months of full dual eligibility preceding admission. Quartiles of number of diagnoses and Elixhauser In-Hospital Mortality Index are among the full sample population. The Elixhauser In-Hospital Mortality Index range includes negative scores due to certain comorbidities being negatively correlated with in-hospital mortality. Quartiles of zip code-level poverty rate are among all US zip code tabulation areas. Zip code-level poverty rate is measured among residents aged >=65.

Source: MedPAR 2021-2022, MBSF 2021-2022, ACS 2019

© 2025 Chatterjee P et al. *JAMA Health Forum*.
