# Supplementary material for: Measurement Bias in Documentation of Social Risk Among Medicare Beneficiaries
Source: JAMA Health Forum. 2025 Jul 18;6(7):e251923. doi: 10.1001/jamahealthforum.2025.1923 (PMC12274977; doi:10.1001/jamahealthforum.2025.1923)
Supplement: Supplement 2. — Data Sharing Statement [file jamahealthforum-e251923-s002.pdf]

Data Sharing Statement

Chatterjee. Measurement Bias in Documentation of Social Risk Among Medicare Beneficiaries. *JAMA Health Forum*. Published July 18, 2025. doi:10.1001/jamahealthforum.2025.1923

Data
Data available: No

Additional Information
Explanation for why data not available: The data for this study can be accessed through independent data use agreements with the Centers of Medicare & Medicaid Services.
